# Supplementary figures and images for: Mitophagy and immune infiltration in vitiligo: evidence from bioinformatics analysis
Source: Front Immunol. 2023 May 23;14:1164124. doi: 10.3389/fimmu.2023.1164124 (PMC10242039; doi:10.3389/fimmu.2023.1164124)

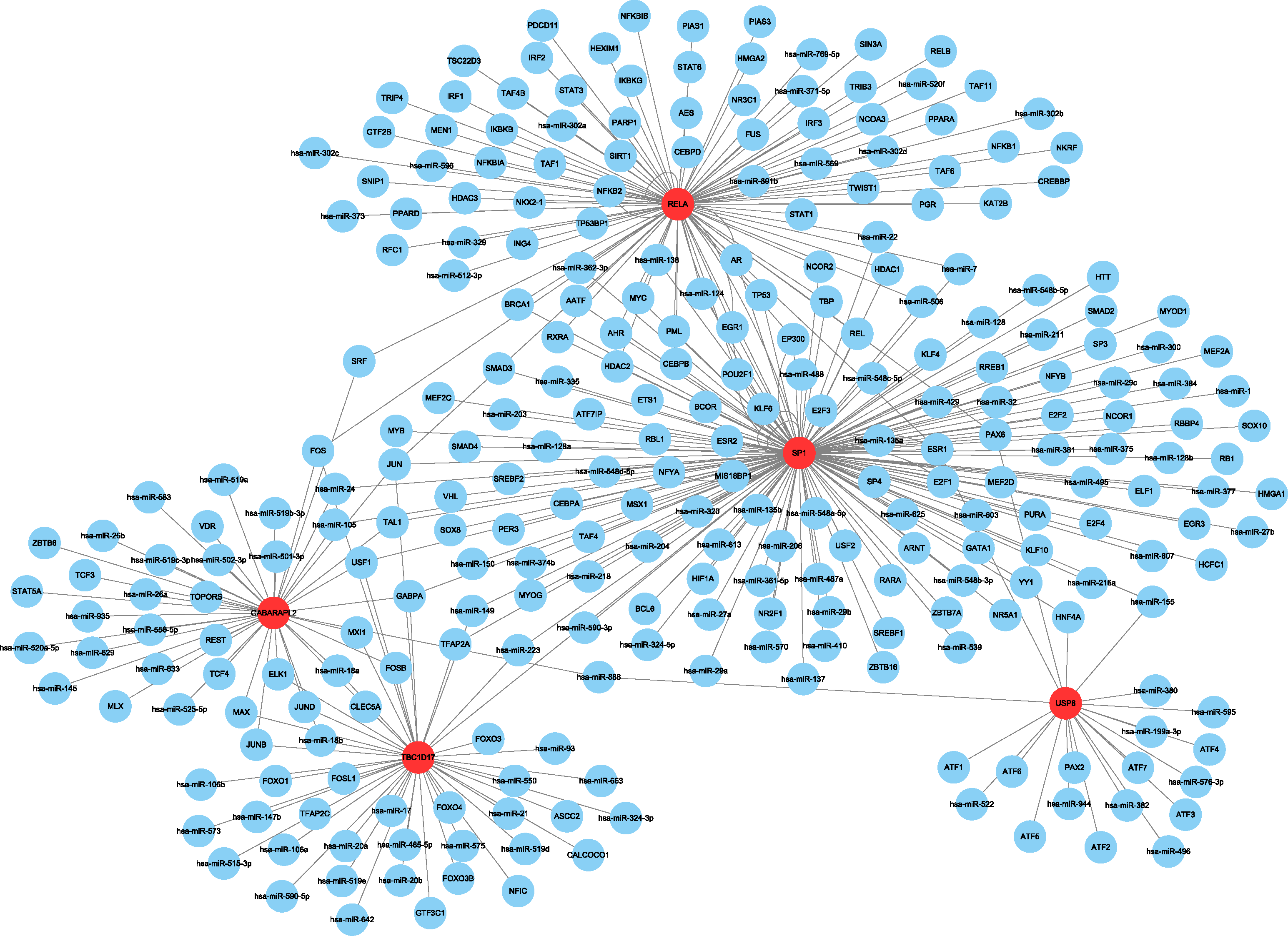

Supplement: Supplementary Figure 1 — TFs and miRNAs regulatory network of hub genes. Upstream regulatory TFs and miRNAs of five mitophagy-related hub genes identified by RegNetwork database [file Image_1.tif]

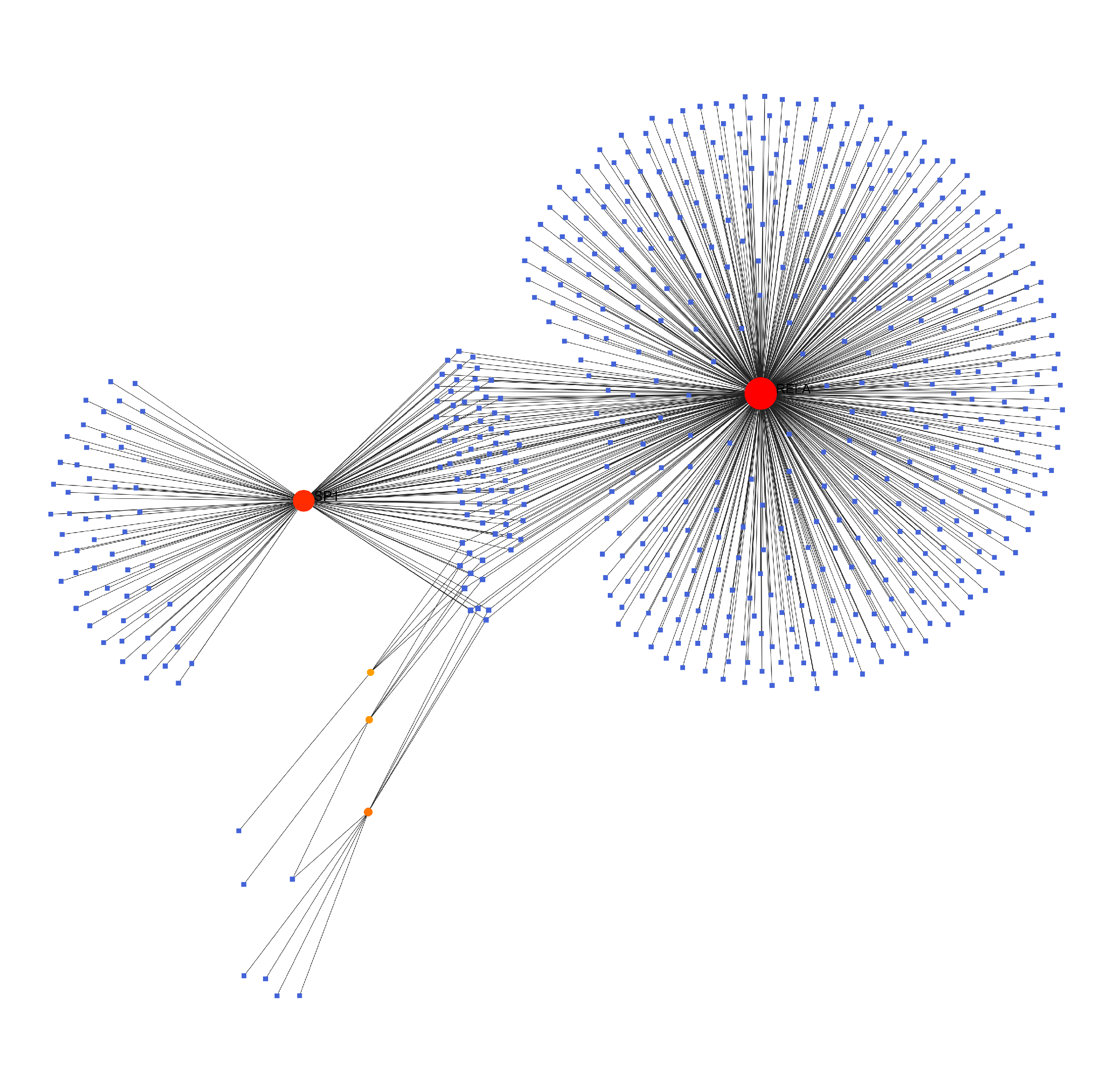

Supplement: Supplementary Figure 2 — Predicted target compounds of hub genes. Protein-compound network of five mitophagy-related hub genes was constructed. [file Image_2.tif]
