# Supplementary material for: Mitophagy and immune infiltration in vitiligo: evidence from bioinformatics analysis
Source: Front Immunol. 2023 May 23;14:1164124. doi: 10.3389/fimmu.2023.1164124 (PMC10242039; doi:10.3389/fimmu.2023.1164124)
Supplement: Supplementary file 3 [file Table_1.docx]

**Supplementary Table S1.** **Primes sequences for qRT-PCR**

|  | *Sequence (5’- 3’)* | *Sequence (5’- 3’)* |
| --- | --- | --- |
| *Primer Set* | *Forward* | *Reverse* |
| GABARAPL2 | CGA GCG AAA TAT CCC GAC AG | TGA TCC ACA TGA ACT GAG CC |
| USP8 | TTCCATTCAATACTTGGACCTGG | CCA AAG AGC CTT TAG CCA ATG T |
| RELA | ATG TGG AGA TCA TTG AGC AGC | CCT GGT CCT GTG TAG CCA TT |
| SP1 | TGG CAG CAG TAC CAA TGG C | CCA GGT AGT CCT GTC AGA ACT T |
| TBC1D17 | GAA CCG GAT CTT CTC GGG G | TTT CTT GCG TAT GTG GGC CTT |
